# Hyperreflective Foci in the Inner Nuclear Layer: Proof‐of‐Concept for an Optical Coherence Tomography Derived Microglia‐Related Marker in Multiple Sclerosis

**DOI:** 10.1002/ana.78215

**Published:** 2026-04-07

**Authors:** Jonathan A. Gernert, Laura M. Bartos, Tara Christmann, Hanna Zausinger, Luca N. Diedrich, Daniel Engels, Miriam Schlüter, Sarah Schlaeger, Sophia Stoecklein, Leonie F. Keidel, Tania Kümpfel, Martin Kerschensteiner, Matthias Brendel, Joachim Havla

**Affiliations:** ^1^ Institute of Clinical Neuroimmunology, LMU Hospital, Ludwig‐Maximilians‐Universität München Munich Germany; ^2^ Biomedical Center, Faculty of Medicine Ludwig‐Maximilians‐Universität München Planegg Germany; ^3^ Department of Nuclear Medicine University Hospital of Munich, LMU Munich Munich Germany; ^4^ Department of Radiology LMU University Hospital, LMU Munich Munich Germany; ^5^ Department of Ophthalmology Ludwig‐Maximilians‐University Munich Germany; ^6^ Munich Cluster for Systems Neurology (SyNergy) Munich Germany; ^7^ DZNE‐German Center for Neurodegenerative Diseases Munich Germany

## Abstract

The role of microglia has emerged as a critical driver of disease progression in multiple sclerosis (MS), but we lack broadly applicable monitoring tools. Here, we investigated whether hyperreflective foci (HRF), as detected by optical coherence tomography (OCT) within the inner nuclear layer (INL) of the retina, can be used as a marker for microglial pathology. We demonstrate that HRF counts are increased in persons with relapsing and progressive MS and correlate with global white and gray matter, as well as deep gray matter [^18^F]GE‐180 uptake.

**Figure:**
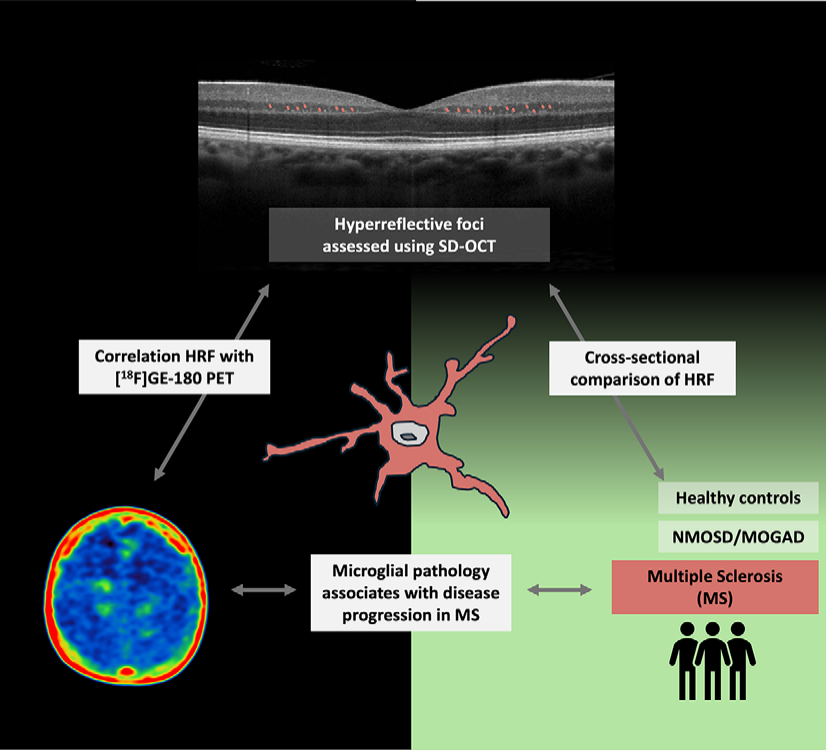
[Color figure can be viewed at www.annalsofneurology.org]

ANN NEUROL 2026;99:1480–1485

[Color figure can be viewed at www.annalsofneurology.org]

Microglial pathology is likely to contribute to progression of relapse‐independent disability in persons with multiple sclerosis (PwMS).^1^ Measuring this microglial status is thus critical for predicting disease trajectories and monitoring emerging therapeutic interventions.^2^ So far, microglial *in vivo* imaging has mainly relied on positron emission tomography (PET) using 18‐kDA translocator protein (TSPO) tracers.^1^ Although these approaches have shown their clinical potential by detecting rim‐active lesions and providing prognostic information on the disease course,^3^, ^4^ their accessibility is limited to highly specialized centers and, due to their radiation exposure, they are not well‐suited for repeated assessments. Therefore, it is important to develop and validate alternative, more broadly applicable methods to assess microglial pathology. One such low‐cost, noninvasive, safe, and easy‐to‐perform method could be optical coherence tomography (OCT) and more specifically the detection of intraretinal hyperreflective foci (HRF) that have been proposed to correlate with other known markers of microglial origin.^5^, ^6^, ^7^ The histopathological origin of HRF in multiple sclerosis (MS) is under debate and no human histopathological data (with OCT correlation) are available in PwMS. However, there are data from individuals with age‐related macular degeneration that show that HRF could also represent melanophages, indicating a histopathological link between HRF and macrophages.^8^

In the present study, we used a robust methodology to count HRF within the inner nuclear layer (HRF_INL_), compared total HRF_INL_ counts among healthy controls (HC), PwMS, and a mixed NMOSD/MOGAD cohort (aquaporin‐4‐IgG‐positive neuromyelitis optica spectrum disorder/myelin oligodendrocyte glycoprotein antibody‐associated disease) and investigated the correlation between total HRF_INL_ counts and [^18^F]GE‐180 uptake in a subgroup of PwMS.

## Methods

The LMU Munich ethics committee approved this study (project number: 25‐0368‐KB) and the German Radiation Protection Committee authorized the study (BfS no. Z 5‐22463/2 2015‐006). We performed a monocentric, retrospective analysis of individuals from the outpatient clinic of the Institute of Clinical Neuroimmunology at LMU Hospital, Munich. This analysis was conducted according to the Declaration of Helsinki.

We compared the HRF_INL_ counts among the following subgroups: (i) relapsing MS (RMS) and progressive MS (PMS) according to Thompson et al^9^; (ii) NMOSD/MOGAD according to Wingerchuk et al^10^ and Banwell et al^11^; and (iii) HC without history of neurological or ophthalmological diseases. Individuals <18 years of age, with > + 5 diopter (dpt), <−5 dpt, diabetes mellitus, ophthalmological diseases, or a clinical history of optic neuritis (ON) were excluded. Expanded Disability Status Scale (EDSS) is reported as score of clinical disability.

OCT scans were performed during November 2014 to December 2023 using a spectral domain OCT (NeuroVisionLab; Spectralis; Heidelberg Engineering GmbH, Heidelberg, Germany; OCT2‐Module). The scan protocol is reported in the Supplementary Material. HRF_INL_ were rated and counted according to current recommendations^12^ by three independent experts blinded to demographic and clinical data using all 25 macula scans. If at least 1 of 25 scan was not suitable for HRF_INL_ analysis, the eye was excluded from further analysis. Total HRF_INL_ counts were calculated as $\sum_{j=1}^{25}{\text{scan}}_{j}$. Individuals with only one eye suitable for analysis were included, and for individuals whose macula scans were evaluated on both sides, the HRF_INL_ mean count was used.

[^18^F]GE‐180 PET scans were performed at the Department of Nuclear Medicine at LMU Hospital, Munich during April 2016 to February 2020, as previously reported.^13^ Magnetic resonance imaging (MRI) scans were performed at the Department of Radiology, LMU Hospital, Munich using a Magnetom Skyra 3T scanner (Siemens, Erlangen, Germany). Please refer to the Supplementary Material for further detailed descriptions on PET and MRI imaging and their analyses.

R software (version 4.4.1; R core team, 2021) was used for statistical analysis. Descriptive statistics are presented as mean ± standard deviation (SD) or median with interquartile range (IQR). The Shapiro–Wilk test was used as test of normality. All statistical tests used are indicated in the Results section in the appropriate place or in the Supplementary Material. Partial correlations adjusted for the time interval between OCT and TSPO‐PET examination (in days) were used to assess associations between HRF_INL_ counts and PET imaging parameters. Statistical significance was set at *p* < 0.05.

## Results

**FIGURE 1:**
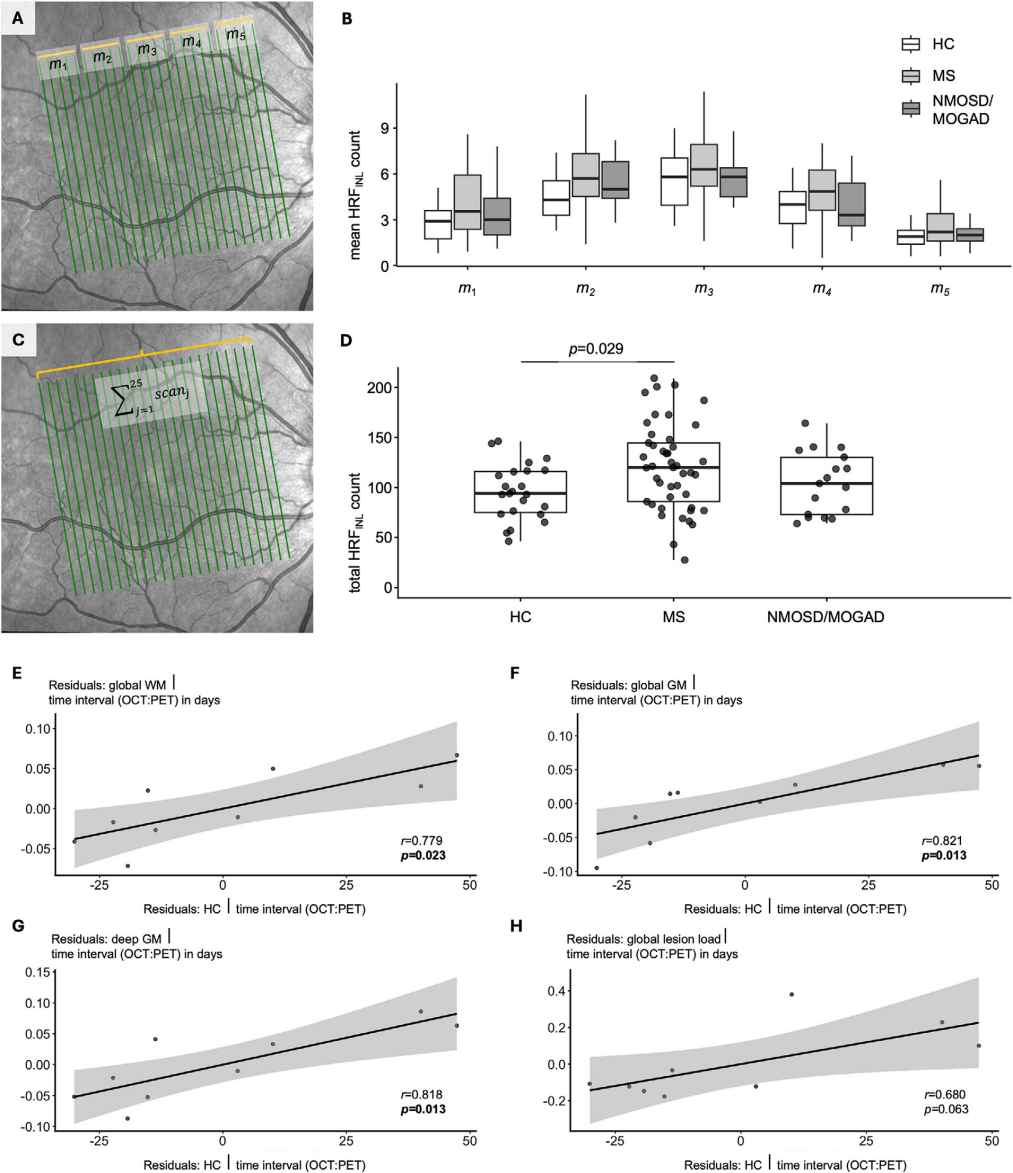
HRF as a potential microglial marker in MS. (A) Macula scan centered to the fovea with 25 B‐scans (*green lines*). HRF were manually counted on each B‐scan. The B‐scans were grouped in 5 sections (*orange lines*) from temporal to nasal (1–5). For each section, mean HRF counts were calculated (*m*_1–5_). (B) Boxplots for mean HRF counts within the 5 sections are displayed for 3 groups (white = healthy controls [HC]; light gray = MS; dark gray = NMOSD/MOGAD. Statistical testing for regional distribution across 3 groups is displayed in the Table 1, comparison analysis within each group is shown in the Supplementary Material. (C) The sum of all HRF was counted across all 25 B‐scans per eye (total HRF_INL_ count). (D) Boxplots for total HRF_INL_ counts (with individual measurements as *black dots*) are displayed for the 3 different groups. Statistical analysis is reported in the Table 1. (E–H) Residual plots for partial correlation between total HRF_INL_ count and SUVr of [^18^F]GE‐180 (corrected for time interval between OCT and PET in days) in global WM (E), global GM (F), deep GM (G), and the global lesional load (H). GM = gray matter; HRF = hyperreflective foci; INL = inner nuclear layer; MOGAD = myelin oligodendrocyte glycoprotein antibody‐associated disease; MS = multiple sclerosis; NMOSD = neuromyelitis optica spectrum disorder; OCT = optical coherence tomography; PET = positron emission tomography; SUVr = referenced standardized uptake values; WM = white matter. [Color figure can be viewed at www.annalsofneurology.org]

Initially, an age‐ and gender‐matched cohort of 131 subjects was screened for HRF_INL_ analysis (Supplementary Material). 85 subjects – where at least one eye with all 25 macula scans was suitable for HRF_INL_ analysis – were included for a cross‐sectional analysis (see the Table). The pattern of regional distribution of HRF_INL_ was similar across HC, PwMS, and patients with NMOSD/MOGAD with highest mean count in the central region (Fig 1A, B; see the Table 1). Using an analysis of covariance (ANCOVA; including gender as cofactor), total HRF_INL_ counts were higher in MS (mean ± SD = 121 ± 43) compared with HC (96 ± 28, *p* = 0.029; Fig 1C, D). Across the three cohorts, the total HRF_INL_ counts did not correlate with the peripapillary retinal nerve fiber layer (pRNFL) thickness (*r* = 0.001, *p* = 0.991) nor the combined macular ganglion cell and inner plexiform layer (mGCIP) volume (*r* = −0.104, *p* = 0.345) but showed moderate association with the macular INL volume (*r* = 0.290, *p* = 0.008; all Pearson). The total HRF_INL_ counts did not differ between patients with RMS (n = 25, 118 ± 45) or PMS (n = 20, 129 ± 36, *p* = 0.374, unpaired *t* test). Across all PwMS, the total HRF_INL_ count was not associated with clinical disability (n = 40, EDSS = 2.6 ± 1.5, *r* = 0.217, *p* = 0.179, Pearson). In an explorative subgroup analysis with 9 PwMS, the referenced standardized uptake values (SUVr) of [^18^F]GE‐180 in the global white (WM) (*r* = 0.779, *p* = 0.023), respectively, global gray matter (GM) (*r* = 0.821, *p* = 0.013) and deep gray matter (*r* = 0.818, *p* = 0.013) correlated with the total HRF_INL_ counts (Fig 1E–H, see the Table 1; and Supplementary Material).

**TABLE 1:** Demographic and Clinical Data

	HC	MS	NMOSD/MOGAD	*p*
Data on cross‐sectional comparison				
Number of subjects, n	23	45	17	
Number of eyes, n	38	75	25	
Gender, Female:Male	14:9	25:20	15:2	**0.050** ^a^
Age, year, median (IQR)	43 (32)	41 (17)	47 (15)	0.867^b^
Disease‐specific information, n		25 RMS	10 NMOSD	
		20 PMS	7 MOGAD	
EDSS, mean ± SD		2.6 ± 1.5	2.5 ± 2.1	0.888^c^
DMT, yes:no		16:29	9:8	0.254^d^
Total HRF_INL_ count, mean ± SD	95.5 ± 27.6	121.0 ± 43.4	104.0 ± 31.2	**0.038** ^e^
m1 HRF_INL_ count, median (IQR)	2.9 (1.9)	3.6 (2.5)	3.0 (2.4)	0.206^f^
m2 HRF_INL_ count, mean ± SD	4.7 ± 1.7	6.0 ± 2.4	5.4 ± 1.7	0.214^g^
m3 HRF_INL_ count, mean ± SD	5.6 ± 1.8	6.5 ± 2.3	6.0 ± 1.8	0.428^g^
m4 HRF_INL_ count, mean ± SD	3.9 ± 1.4	5.0 ± 2.0	4.0 ± 1.7	0.081^g^
m5 HRF_INL_ count, median (IQR)	1.9 (0.9)	2.3 (1.8)	2.0 (0.8)	0.141^f^
Data on PwMS subgroup with TSPO‐PET				
Number of subjects, N		9		
Number of eyes, n		17		
Gender, Female:Male		3:6		
Age, year, median (IQR)		49 (15)		
Disease course, N		3 RMS		
		6 PMS		
EDSS, median (IQR)		3.0 (0.75)^h^		
DMT, yes:no		5:4		
Time interval OCT and TSPO‐PET examination, days, median (IQR)		81 (65)		
SNP status		4 MAB		
		5 HAB		

Bold values are statistically significance *p* < 0.05.

^a^ Fisher's Exact test with *p* = 0.04994. Pairwise comparison using Benjamini–Hochberg: HC vs MS *p* = 0.797; HC vs NMOSD/MOGAD *p* = 0.115; MS vs NMOSD/MOGAD *p* = 0.0567. For further comparisons among the 3 groups, gender was considered as a cofactor due to the (marginal) differences in gender distribution.

^b^ Kruskal‐Wallis test not significant.

^c^ ANOVA test not significant.

^d^ Fisher's Exact test not significant.

^e^ ANCOVA using gender as cofactor with Bonferroni adjustment: HC vs MS *p* = 0.029; HC vs NMOSD/MOGAD *p* = 0.329; MS vs NMOSD/MOGAD *p* = 0.775.

^f^ Kruskal‐Wallis test, because the variable was not normally distributed.

^g^ ANCOVA using gender as cofactor.

^h^ EDSS not available for 2 subjects (N = 7).

DMT = disease modifying therapy; EDSS = expanded disability status scale; HAB = high‐affinity binding; HC = healthy controls; HRF = hyperreflective foci (absolute counts, please also refer to the Figure); INL = inner nuclear layer; IQR = interquartile range; MAB = medium‐affinity binding; MS = multiple sclerosis; NMOSD = aquaporin 4 antibody‐positive neuromyelitis optica spectrum disorder; MOGAD = myelin oligodendrocyte glycoprotein antibody disease; OCT = optical coherence tomography; PMS = progressive multiple sclerosis; Pw = persons with; RMS = relapsing multiple sclerosis; SD = standard deviation; SNP = single‐nucleotide polymorphism (rs6971‐SNP); TSPO‐PET = positron‐emission‐tomography (PET) scans using the radiotracer [^18^F]GE‐180; n = number

## Discussion

Compared with previous studies on HRF in PwMS, which counted HRF based on a single macular B‐scan,^5^, ^6^, ^7^, ^14^ we chose a broader approach by counting the HRF in 25 macula B‐scans per eye to minimize the potential impact of possible outliers. With this robust counting method, we were able to trace the regional distribution of HRF_INL_. Interestingly, we demonstrated that HRF_INL_ are predominantly found in the central area and thus less in the peripheral areas in all 3 groups studied (HC, MS, and NMOSD/MOGAD). Furthermore, our results are consistent with published analyses showing a trend toward higher HRF_INL_ in individuals with MS compared to HC.^5^, ^6^, ^7^, ^14^ In our subgroup analysis, we found comparable HRF_INL_ scores regardless of the disease course of MS. In contrast to our findings, a recent study reported elevated HRF_INL_ values not only in MS but also in NMOSD.^14^ These differences may be explained by the following considerations: (i) we included individuals with PMS, respectively, MOGAD; (ii) we excluded eyes with prior ON to reduce possible confounding effects on HRF_INL_ counts; and (iii) additionally, immunotherapies might influence the HRF_INL_ counts. Such effects have been shown, for example, for PwMS.^15^ Our results discussed here, are limited by its cross‐sectional approach. In the future, multicenter studies with longitudinal OCT examinations are needed to investigate the most reliable and time‐effective method for counting HRF. The temporal course of HRF counts after acute ON (in MS, NMOSD, and MOGAD) need to be further studied. In addition, HRF counts in other retinal layers are of high interest since increased HRF counts in the mGCIP and outer nuclear layer (ONL) have been reported in PwMS.^14^, ^16^

The histopathological correlate of HRF in MS is under debate, since, as far as we know, no human comparative studies (OCT and immunohistochemistry) have been published yet. A complicating factor is that HRF are not a static finding, but rather a dynamic one. In principle, previous histopathological data in MS suggest that HLA‐DR‐positive cells can be found in the INL (and in perivascular areas).^17^ On the other hand, OCT examinations in PwMS (especially after excluding individuals with prior ON) demonstrated a correlation with atrophy rates of the global WM, GM, and specific deep GM structures.^18^ Therefore, it has been repeatedly concluded that OCT can visualize and monitor global brain pathologies in PwMS. Based on these considerations and data from other ophthalmological diseases, we conducted a correlation analysis between HRF and TSPO‐PET. We demonstrated high correlations between HRF and global [^18^F]GE‐180 uptake in WM and GM in a subgroup analysis. The correlation between HRF and [^18^F]GE‐180 uptake in cerebral lesions was borderline but showed a clear trend. Both local (lesion‐associated) and global (predominantly in progressive MS) increased TSPO‐PET signals have been repeatedly reported.^19^ In summary, we add further evidence that HRF_INL_ could be a potential marker for microglia‐related pathology, which is thought to be involved as a possible driver of relapse‐independent progression in PwMS. However, we would also like to emphasize that the generalizability of our subgroup analysis is limited due to the small number of cases, the heterogeneity of the study, and, not least, the retrospective nature of the analysis. Our results therefore require external validation in larger cohorts, ideally including MRI data (for assessment of paramagnetic rim lesions), and extending the use of other TSPO tracers. Nevertheless, these proof‐of‐concept data provide evidence that OCT‐based detection of HRF_INL_ could serve as a widely accessible, versatile microglia‐related marker in PwMS and underscore the importance of retinal imaging in PwMS.

## Author Contributions

JAG, MK, MB, and JH contributed to the conception and design of the study; JAG, LMB, TC, HZ, LD, DE, MS, SaS, SoS, LFK, MB, and JH contributed to the acquisition and analysis of data; JAG, LMB, TK, MK, MB, and JH contributed to drafting the text or preparing the figures.

## Potential Conflicts of Interest

The authors have nothing to report.

## Supporting information


**Supplementary Data S1.** Supporting Information.

## Data Availability

The data of this study are available upon reasonable request to the corresponding author.
