# Supplementary material for: Hyperreflective Foci in the Inner Nuclear Layer: Proof‐of‐Concept for an Optical Coherence Tomography Derived Microglia‐Related Marker in Multiple Sclerosis
Source: Ann Neurol. 2026 Apr 7;99(6):1480–5. doi: 10.1002/ana.78215 (PMC13206537; doi:10.1002/ana.78215)
Supplement: Supplementary file 1 — Supplementary Data S1. Supporting Information. [file ANA-99-1480-s001.docx]

**Supplementary Material**

1. **Additional Methods information**

1.1 Spectral domain-optical coherence tomography (SD-OCT)

An SD-OCT device (Spectralis®; Heidelberg Engineering GmbH, Heidelberg, Germany; OCT2-Module) was used for B-scan acquisition. Macula SD-OCT scans were acquired using the following protocol: 25 vertical B-scans (30x25°). Heyex v2.5.5 was used for automated retinal layer segmentation. The volume (mm^3^) of the combined ganglion cell an inner plexiform layer (GCIP) and inner nuclear layer (INL) derived from a 3mm ring centered to the fovea. Peripapillary retinal nerve fiber layer (pRNFL) thickness (µm) was measured using a 3mm ring scan centered on the optic nerve head.

1.2 [^18^F]GE-180 positron emission tomography (TSPO-PET)

Only patients who had OCT and PET scans within <180 days time interval were included. In short, patients were scanned using a Biograph 64 PET/CT (Siemens, Erlangen, Germany). First, a low-dose CT scan was performed for attenuation correction. Second, patients were dynamically scanned over 90min after intravenous injection of 189 ± 12 MBq [^18^F]GE-180 and the late acquisition phase (60-90 min p.i., single frame) was used for analysis. A matrix size of 256 × 256 × 109, and a voxel size of 1.336 × 1.336 × 2.027 mm was used for 2D image reconstruction. All TSPO-PET image analyses were performed by PMOD (version 3.5/version 4.3, PMOD Technologies). Spatial normalization of TSPO-PET and MRI images to the atlas space was performed to allow standardized analyses across patients. Region-based partial volume correction was added to account for potential brain atrophy in PwMS. The Hammers atlas served to delineate tracer uptake in different brain regions in each patient ^[[1]](#footnote-1)^: global white matter (WM), global gray matter (GM), deep GM and specific GM (please also refer to the **Supplement Table**). TSPO-PET images were intensity normalized to the mean TSPO-PET signal of the cerebellar cortex to obtain maps of relative regional TSPO tracer uptake as standardized uptake value ratios (SUVr). Lesional TSPO-PET SUVr was determined within white matter lesions segmented via MRI (see below). Genotyping for the TSPO binding status was performed in all 9 patients at the Department of Psychiatry of the University Hospital LMU Munich and Regensburg. Genomic DNA was extracted from whole blood using a SQ Blood DNA kit von Omega Bio-Tek (Norcross, GA, USA) according to the manufacturer’s protocol. DNA quality was assessed by optical absorbance and gel electrophoresis. TaqMan quantitative polymerase chain reaction assays were used for amplification, Sager method for sequencing. Only medium- and high-affinity binders (MAB, HAB) were included, as previous reports showed no significant difference in tracer binding between both genotypes but lower binding capacity in low-affinity binders (LAB) ^[[2]](#footnote-2)^. One patient was a priori excluded due to low-affinity binding (LAB) status.

1.3 Magnetic resonance imaging (MRI)

Following sequences were acquired: axial T_2_-weighted, T_2_-FLAIR, T_1_-weighted, and post-contrast T1-weighted images (3mm slice thickness). Lesion maps were created using the LST toolbox ^[[3]](#footnote-3)^ to assess lesional [^18^F]GE-180 uptake: White matter lesions were segmented based on T_2_-FLAIR sequences using the lesion prediction algorithm (Schmidt, 2017, Chapter 6.1) as implemented in the LST toolbox version 3.0.0 (statistical-modelling.de/lst.html).

1. **Additional Results information**

2.1 Screening

Initially, 25 macular scans per eye were evaluated for HRF_INL_ in an age- and gender-matched cohort of 209 eyes from 131 individuals (31 HC, 73 MS, 27 NMOSD/MOGAD), considering the inclusion and exclusion criteria (total of 5,225 B-scans). Eyes were then excluded in which at least one B-scan was not suitable for the detection of HRF, and 138 eyes from 85 individuals were included in the final analysis.

2.2 Distribution of HRF_INL_ across the macula

The mean HRF_INL_ count of the five different segments (*m_1-5_*) shown in **Figure A** were calculated and compared within the three different subgroups (HC, MS and NMOSD/MOGAD) using an ANOVA with Bonferroni-adjustment (**Supplement Figure**):

$\boldsymbol{m}_{\boldsymbol{1}}=\frac{1}{5}\sum_{j=1}^{5} {HRF count}_{image j}$, $\boldsymbol{m}_{\boldsymbol{2}}=\frac{1}{5}\sum_{j=6}^{10} {HRF count}_{image j}$, *etc.*


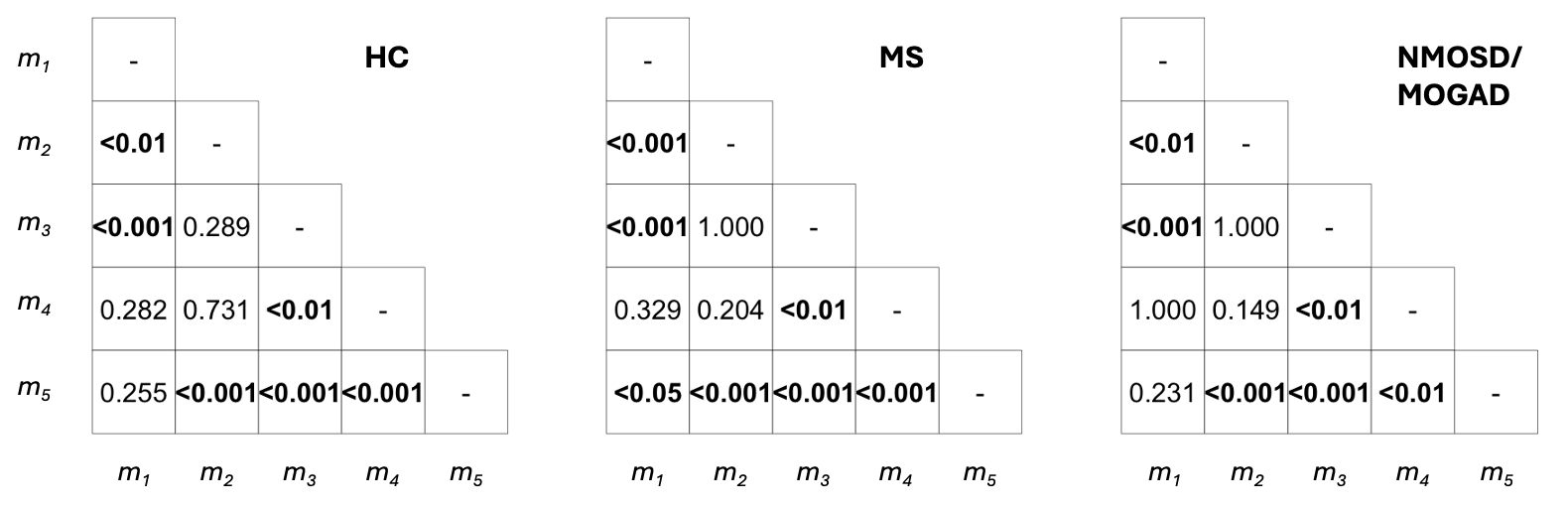


**Supplement Figure**

ANOVA with Bonferroni-adjustment comparing *m_1-5_* within HC, MS and NMOSD/MOGAD.

2.3 Partial correlation between OCT and TSPO-PET

Partial correlations between total HRF_INL_ counts and referenced standardized uptake values (SUVr) in different brain regions were calculated, considering the time interval between OCT and TSPO-PET examination (in days) (**Supplement Table**).

|  | *r*-value | *p*-value |
| --- | --- | --- |
| global WM | 0.779 | **0.023** |
| global GM | 0.821 | **0.013** |
| deep GM | 0.818 | **0.013** |
| - Amygdala | 0.735 | **0.038** |
| - Caudate Ncl. | 0.662 | 0.074 |
| - Hippocampus | 0.293 | 0.482 |
| - Putamen | 0.650 | 0.081 |
| - Thalamus | 0.638 | 0.089 |
| global lesion load | 0.680 | 0.063 |

**Supplement Table**

Correlation analysis (Pearson) between total HRF_INL_ count and SUVr in different brain regions in n=9 PwMS. Regions considered for deep GM in alphabetic order: Amygdala, Caudate Nucleus, Hippocampus, Putamen, Thalamus. GM: gray matter; WM: white matter.

1. Fan, L., et al., *The Human Brainnetome Atlas: A New Brain Atlas Based on Connectional Architecture.* Cereb Cortex, 2016. **26**(8): p. 3508-26. [↑](#footnote-ref-1)
2. Vettermann, F.J., et al., *Impact of TSPO Receptor Polymorphism on [(18)F]GE-180 Binding in Healthy Brain and Pseudo-Reference Regions of Neurooncological and Neurodegenerative Disorders.* Life (Basel), 2021. **11**(6). [↑](#footnote-ref-2)
3. Schmidt, Paul et al. “Automated segmentation of changes in FLAIR-hyperintense white matter lesions in multiple sclerosis on serial magnetic resonance imaging.” *NeuroImage. Clinical* vol. 23 (2019): 101849. doi:10.1016/j.nicl.2019.101849 [↑](#footnote-ref-3)
